# Post-traumatic stress disorder (PTSD) and associated factors in the population victim of violence in Cordoba, Colombia

**DOI:** 10.1192/j.eurpsy.2023.2081

**Published:** 2023-07-19

**Authors:** K. Cabas-Hoyos, I. Villamil-Benitez, A. Uribe-Urzola

**Affiliations:** 1 Grupo Cognición y Educación, Universidad del Magdalena, SANTA MARTA; 2 Faculty of Psychology, Universidad Pontificia Bolivariana, Monteria, Colombia

## Abstract

**Introduction:**

The Department of Cordoba is located in the north of Colombia and is a key territory for understanding the political violence that the country has experienced. Cordoba has suffered the armed conflict with various groups present in the area. In the category of victimizing acts, more than 30,000 victims of forced displacement have been reported and 4,621 for forced disappearance.

**Objectives:**

The objective of this study is to evaluate the prevalence of PTSD and associated factors in a population victim of violence in Cordoba, Colombia.

**Methods:**

Design: cross-sectional, using quantitative data. Participants: victims of forced displacement in Cordoba (n=95), of whom 42.75% (n=45) were men and 57.25% (n=50) were women. The mean age of the participants was 40.7 (SD 14.1) Instruments: Clinician-Administered PTSD Scale (CAPS) (Blake et al., 1990) was administered to check clinical symptoms of the Disorder; to assess the causes of symptoms was administered The PTSD Checklist PCL-C is a short version of the PTSD Checklist – Civilian version (Weathers, 1993). Additionally, the BDI was administered to assess depression and STAI to measure anxiety. Data analysis: a Student’s t-test was used to assess the difference by gender and age groups. Descriptive statistics were also used to identify clinical characteristics of the sample.

**Results:**

In relation to the activation factor, it was found that the men in the sample presented significant differences compared to the women [Activation. F= (1.117) = .79; p = 0.00]. Meanwhile, PTSD was presented equally in both sexes and in all age groups with a prevalence of 26% of the sample. The most prevalent events assumed to cause the disorder were extreme human suffering and natural disaster. Depression levels were moderate in 33.3% of the population and state anxiety showed a level of 77.8% of the sample.

**Conclusions:**

Our study finds that 26% of the population suffered from PTSD and in a significant percentage comorbidities were found with depression, anxiety, added to the vulnerability of those who have experienced these events, that is, revictimization, low access to social services, low schooling and poverty. It is important to consider the multifactorial nature of PTSD and its relationship with the presence of traumatic events (Bados, 2015; Kessler et al., 2014). In Colombia there is a challenge related to the intervention of this population, which constitutes future lines in our research.

**Disclosure of Interest:**

None Declared

